# BONE-Net: A novel hybrid deep-learning model for effective osteoporosis detection

**DOI:** 10.1371/journal.pone.0334664

**Published:** 2025-10-16

**Authors:** Ishaq Muhammad, Routhu Srinivasa Rao, Bumshik Lee

**Affiliations:** 1 Department of Information and Communication Engineering, Chosun University, Gwangju, Korea; 2 Energy AI, Korea Institute of Energy Technology (KENTECH), Naju, Korea; Politecnico di Torino, ITALY

## Abstract

Osteoporosis is a prevalent bone disease characterized by reduced bone density and an elevated risk of fractures, especially in older adults and postmenopausal women. The clinical consequences of osteoporotic fractures extend beyond pain and disability, contributing substantially to morbidity, mortality, and healthcare costs. Early intervention and accurate detection is therefore essential to improve patient outcomes. This paper introduces an advanced deep-learning methodology to enhance the accuracy and efficiency of osteoporosis detection through knee X-ray analysis. The proposed approach integrates features from two pre-trained models, DenseNet169 and Vision Transformer (ViT), with a custom-designed Attention Model (AM) to capture detailed spatial and channel-specific information from the input images. These fused features are then fed into a fully connected neural network to classify the images as osteoporotic or normal. The results indicate significant improvements in classification accuracy, achieving a high accuracy rate on previously unseen test data. The proposed model achieves superior performance over existing methods and other recent models for osteoporosis detection, with an accuracy of 0.8611, specificity of 0.9474, and precision of 0.9286. Our approach effectively combines convolutional and transformer-based representations, enabling extraction of both local and global features for comprehensive bone characterization. These findings highlight the model’s potential to support early diagnosis, timely intervention, and improved patient care in osteoporosis management.

## Introduction

Human osteoporosis is a pervasive and debilitating bone disease characterized by decreased bone mineral density (BMD) and a heightened risk of fractures [1]. It primarily affects older adults, especially postmenopausal women, leading to significant morbidity and mortality [2,3]. The clinical significance of osteoporosis stems from its potential to cause fractures, particularly in the hip, spine, and wrist, which can result in chronic pain, disability, and even death [4,5]. As the global population ages, the incidence of osteoporosis and related fractures is expected to rise, highlighting the urgent need for effective diagnostic and therapeutic strategies. Early detection and diagnosis of osteoporosis are crucial for preventing fractures and their associated complication [6]. Traditional methods for assessing BMD, such as dual-energy X-ray absorptiometry (DXA), are considered the gold standard but are often limited by issues of accessibility, cost, and radiation exposure [7]. Moreover, these methods may not fully capture the complex structural changes in bone architecture associated with osteoporosis. According to [8], osteoporosis affects approximately 200 million people worldwide. In the United States, more than 10 million individuals are projected to have osteoporosis, with an additional 44 million having low bone mass, which increases their risk [9]. The economic burden of osteoporosis-related fractures is significant, with annual costs estimated to exceed $20 billion in the United States [9]. Therefore, there is an urgent need for more advanced, accurate, and accessible methods for early detection and monitoring of osteoporosis.

Recent advancements in artificial intelligence, especially deep-learning, have shown promise in transforming medical diagnostics, including the detection and analysis of osteoporosis. Deep-learning models, a subset of machine learning, can automatically learn and extract features from large datasets, making them well-suited for analyzing medical images and other complex data. In the context of osteoporosis, deep-learning approaches can be used for various tasks, such as estimating BMD, segmenting bone structures, predicting fracture risk, classifying osteoporosis severity, and detecting abnormalities in bone images. Applying deep-learning to osteoporosis research offers significant promise for enhancing the accuracy and efficiency of diagnosis and management. By utilizing large datasets and advanced algorithms, these methods can provide more detailed and reliable assessments of bone health, leading to improved patient outcomes. Recent advancements in deep-learning techniques have demonstrated remarkable potential in osteoporosis management, including automated BMD estimation from medical images, fracture risk prediction, and bone segmentation [10,11].

Furthermore, deep-learning models can detect subtle changes in bone microarchitecture that may not be visible to the human eye, potentially enabling earlier diagnosis and intervention. These models can also integrate various data types, including imaging, clinical, and genetic information, to provide a comprehensive assessment of bone health and fracture risk.

### Characteristics of osteoporosis in radiograph

**Fig 1:**
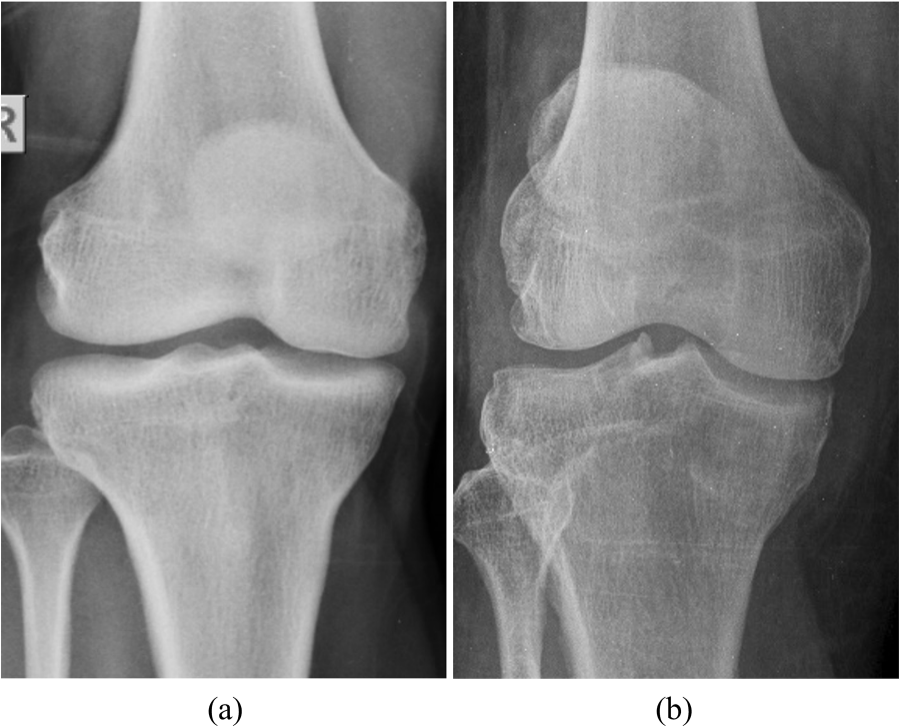
Normal image and osteoporotic image.

Osteoporotic changes in the knee requires a keen understanding of several visual cues. Fig 1 illustrates normal and osteoporotic images. In Fig 1(a), normal bone structures, especially in the femoral and tibial regions, appear denser with well-defined trabecular patterns and sharp cortical boundaries in a normal knee radiograph. Additionally, the cortical bone, the dense outer surface, is thicker with a more distinct boundary in a healthy knee. The joints are well-aligned, and the space between the bones is typically uniform, indicating healthy cartilage. In contrast, Fig 1(b) shows an osteoporotic knee with markedly altered characteristics. The most notable feature is the reduction in BMD, leading to increased radiolucency, where bones appear lighter or more translucent. The cortical bone is thinner and less defined, contributing to a “washed-out” appearance. The trabecular pattern also changes significantly there may be fewer trabeculae, or the pattern may appear disorganized and less dense. This loss of trabecular connectivity weakens the internal bone structure, a hallmark of osteoporosis. Additionally, micro-cracks and fractures can develop more easily in osteoporotic bones due to their increased fragility. These micro-cracks may not be visible in the early stages but can be inferred from subtle changes in bone architecture, such as irregularities in trabecular patterns or localized areas of increased radiolucency. Key signs of osteoporosis in a radiograph include cortical thinning, disorganized or sparse trabecular patterns, and indications of micro-cracks or fractures. It is also important to assess any asymmetry in joint spaces, as this may indicate uneven bone wear due to weakened structural integrity. Given the subtlety of early-stage osteoporosis, developing a classification system that detects variations particularly changes in bone texture, trabecular alignment, and cortical integrity is essential for improving diagnostic accuracy and enabling early intervention.

This study applies deep-learning to detect abnormalities in the knee osteoporotic (KOP) dataset [12] to develop and evaluate our approach. By focusing on the knee, an area which is often affected by osteoporotic fractures and is underexplored, the approach aims to use these radiographic characteristics for accurate and automated identification of osteoporotic changes. The main contribution of the works are as follows:

- We propose a novel deep-learning approach that combines DenseNet169 [13] and Vision Transformer (ViT) [14] models with an Attention Model (AM) for enhanced osteoporosis detection.
- A custom AM is designed, incorporating channel and spatial attention mechanisms to capture detailed spatial and channel-specific information from input images.
- Combining features from both models and the AM produces a comprehensive representation of input images, resulting in more accurate classification.
- The Fully Connected (FC) neural network is carefully tuned through extensive experimentation to enhance the model’s overall performance.

## Related works

Medical imaging has been advancing rapidly, with AI playing a crucial role in various applications such as image segmentation, natural language processing, classification [15–18]. These advancements have enhanced diagnostic accuracy, improved efficiency, and expanded the capabilities of automated medical analysis. In particular, AI-driven techniques are being increasingly utilized to assist in the detection and diagnosis of complex diseases, such as osteoporosis enabling earlier intervention and more effective treatment strategies. With the rising prevalence of osteoporosis, early diagnosis and effective treatment are essential for improving medical imaging applications and reducing fracture risk. [8,19]. Significant advancements in medical imaging and machine learning have improved early detection and diagnosis of osteoporosis. This section reviews state-of-the-art (SOTA) methodologies and highlights key findings from the relevant literature.

### Osteoporosis detection techniques

The detection of osteoporosis has advanced significantly with the development of advanced medical imaging and deep-learning techniques to improve diagnostic accuracy. While traditional methods like DXA remain the gold standard, they have limitations, including low availability, high cost, and radiation exposure [20,21]. Recent advancements use deep-learning models to enable automatic detection from clinical data and medical images, enhancing diagnostic precision and efficiency [20].

### Screening techniques

Screening for osteoporosis typically involves assessing BMD and other risk factors. DXA continues to be the most reliable method for assessing BMD. However, recent research has explored more accessible screening methods [22]. Wang et al [23] proposed a fully automated deep-learning system for osteoporosis screening using chest Computed Tomography (CT) images. The authors developed an accurate vertebral bone segmentation model with a high Dice coefficient and implemented DenseNet and ResNet-18 [24] architectures for BMD classification. These models demonstrated excellent diagnostic performance, enabling efficient and cost-effective osteoporosis screening without additional radiation exposure [23].

### Prediction techniques

Deep-learning models can predict the risk of osteoporosis and related fractures by analyzing various risk factors, including demographic data, clinical history, and genetic information. For example, a study developed a machine learning predictive model for osteoporosis risk using chronic disease data from 10,000 patients in Germany [25], This study employed nine different algorithms for feature selection and created a stacked ensemble model that combined logistic regression [26], AdaBoost [27], and Gradient Boosting [28] techniques. The model achieved an AUC-ROC of 0.76, indicating good predictive performance. SHAP [29] values were used to assess feature importance, identifying age, gender, and chronic diseases as key predictors. This approach aims to facilitate early identification and intervention for high-risk individuals in healthcare settings. Another work by Tu et al. [30] developed a deep-learning model to predict osteoporosis using simple hip radiographs. Using 1001 matched DXA and radiographic images, a VGG16-based deep neural network was trained and validated. The model achieved 81.2% accuracy, with high sensitivity (91.1%) and an AUC of 0.867. External validation confirmed its reliability, highlighting its potential as a practical osteoporosis screening tool in clinical settings.

### Bone fracture detection

Bone fracture detection, a critical aspect of osteoporosis management, has significantly improved with artificial intelligence. Automated systems using deep-learning can identify abnormalities in bone images, enabling early diagnosis and intervention. Several studies have explored the use of deep-learning for osteoporosis detection and management. CNNs [31] have been particularly effective in analyzing medical images [32] [33]. For instance, CNNs have been used to automatically estimate BMD from radiographs and CT scans with high accuracy. Other studies have developed deep-learning models for segmenting trabecular and cortical bone, which are critical for assessing bone quality [34]. Predictive models based on clinical and imaging data have also been created to estimate fracture risk [35]. In [36], a deep-learning model was introduced to detect vertebral fractures and osteoporosis on lateral spine radiographs, demonstrating high sensitivity and specificity. This approach could lead to earlier detection of osteoporotic fractures, which are often missed in routine examinations. Incorporating these deep-learning scores with clinical indications improved the accuracy of referrals for bone-density testing in high-risk individuals.

### Classification techniques

Classification of osteoporosis severity and risk has recently been a focus of research, with several notable studies employing advanced machine learning techniques. Here, we discuss some of the recent works that have made significant contributions to this field: In [37], a deep-learning model named Osteo-Net was proposed for diagnosing osteoporosis from knee X-ray images. The model uses deep neural networks to extract high-level features from low-quality X-ray images, achieving an accuracy of 82.61%. Osteo-Net is presented as a low-cost and computationally efficient alternative for early osteoporosis diagnosis, especially useful in primary healthcare centers without DXA machines. The study highlights the model’s superior performance compared to other SOTA methods and suggests its potential for mass screening and preventive care. Another study by [38] proposed a deep-learning model for osteoporosis detection from knee X-rays that combines multi-level feature fusion, and dual attention mechanisms. Experiments show improved classification performance and reduced false positives compared to existing methods. In [39], a new approach for assessing BMD from CT scans was proposed using a U-Net [40] architecture with a VGG11 encoder. This methodology, trained on 24,318 image pairs, automatically places regions of interest (ROIs) in the L1 vertebral body, simulating manual measurements. The model demonstrated improved ROI placement and robustness across various patient positions. It facilitates opportunistic osteoporosis screening by leveraging routine CT scans, enhancing sensitivity and specificity for a broader, cost-effective osteoporosis assessment. Kang *et al.* [41] discusses using a deep-learning model, ResUNet-a [42], to predict BMD and fracture risk from clinical data and CT images. The model employs semantic segmentation for ROI extraction and integrates clinical data to improve prediction accuracy. This approach offers a promising method for non-invasive osteoporosis screening and fracture risk assessment. Xue *et al.* [43] introduced an approach using CoTNet [44] with fusion blocks to create a model called FcoTNet. Their work integrates a multi-scale weighted fusion module into a transformer-style architecture to enhance the extraction of texture and structural features. In experiments on an internal dataset, FcoTNet outperformed other SOTA (SOTA) classification networks. The model also demonstrated improved performance compared to clinicians in internal medical tests and provided valuable assistance in diagnosis. In [45], an ensemble learning approach for knee osteoporosis detection, named KONet, was proposed. This model combines two deep-learning models, EfficientNetB0 [46] and DenseNet121 [13], to extract and fuse critical features, enhancing accuracy, sensitivity, and specificity compared to individual models. KONet outperformed several SOTA methods, demonstrating its potential as a valuable tool for aiding clinicians in diagnosing knee osteoporosis. The current research on osteoporosis detection reveals several critical gaps that our work aims to address. Many existing studies rely heavily on clinical covariates to improve diagnostic accuracy, whereas our approach focuses on a direct and scalable imaging-based solution. In addition, transformer-based models remain underexplored in osteoporosis detection despite their success in other areas; we address this by integrating a ViT into our architecture to capture global contextual relationships within images. Moreover, research specifically targeting knee osteoporosis is limited, despite its clinical significance. Our study fills this gap by concentrating on knee X-ray images, providing a more focused and clinically relevant analysis. Finally, robust multi-scale feature extraction techniques are lacking in the literature, restricting models’ ability to detect subtle textural changes indicative of early-stage osteoporosis. We overcome this limitation by incorporating an AM with weighted fusion, thereby enhancing the robustness and sensitivity of the diagnostic model.

## Materials and methods

The proposed methodology aims to use advanced deep-learning techniques to significantly enhance the accuracy and efficiency of osteoporosis detection and classification using the KOP dataset. This innovative approach addresses the limitations of traditional diagnostic methods by employing SOTA deep-learning techniques. The model architecture includes several critical stages: data preparation, feature extraction using pre-trained models, implementation of the AM, feature fusion, and binary classification. Each stage is essential for developing a robust and reliable system for early osteoporosis detection.

### Data preparation and pre-processing

**Fig 2:**
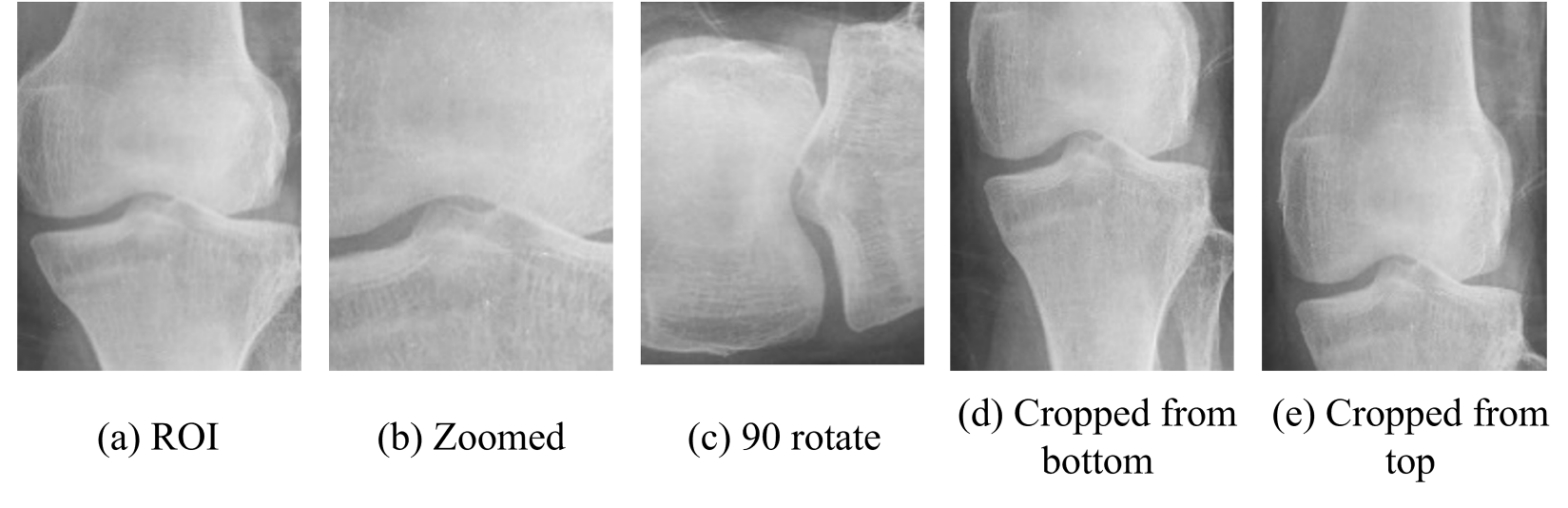
Samples of augmented images.

The data preparation stage involves sourcing and augmenting the dataset to enhance model performance. The dataset, obtained from Kaggle, consists of 372 grayscale X-ray images stored in PNG format, with an original resolution of 1060 × 2660 pixels. These images, which include 186 normal and 186 osteoporotic cases, are first processed by cropping the ROIs to obtain images with a resolution of approximately 300 × 450 pixels. For the experiments, these images are further resized to 224 × 224 pixels. The dataset is divided into three subsets: 300 images for training, a validation set derived from the training data with an 80:20 split, and 72 non-augmented, unseen images reserved for testing. To improve model robustness and reduce overfitting, each training image undergoes augmentation to generate five additional versions, resulting in a total of 1800 images: specifically, 1440 images for training and 360 images for validation. The augmentation techniques applied include zooming the image by 1.5× and 2 × , cropping from the top at a 75:25 ratio and from the bottom with the same ratio, and rotating the image by 90 degrees. These augmentations enhance the model’s ability by diversifying the training examples, as illustrated in Fig 2. The testing set, consisting of 72 unseen and untouched images, is reserved for the final evaluation of the model.

### Feature extraction

**Fig 3:**
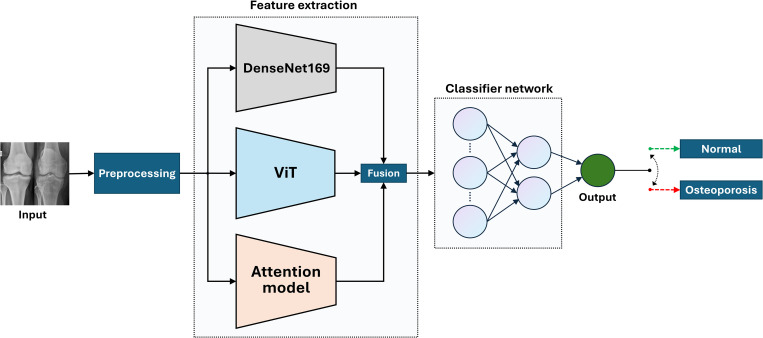
Illustration of the proposed model for osteoporosis detection using the KOP dataset.

**Fig 4:**
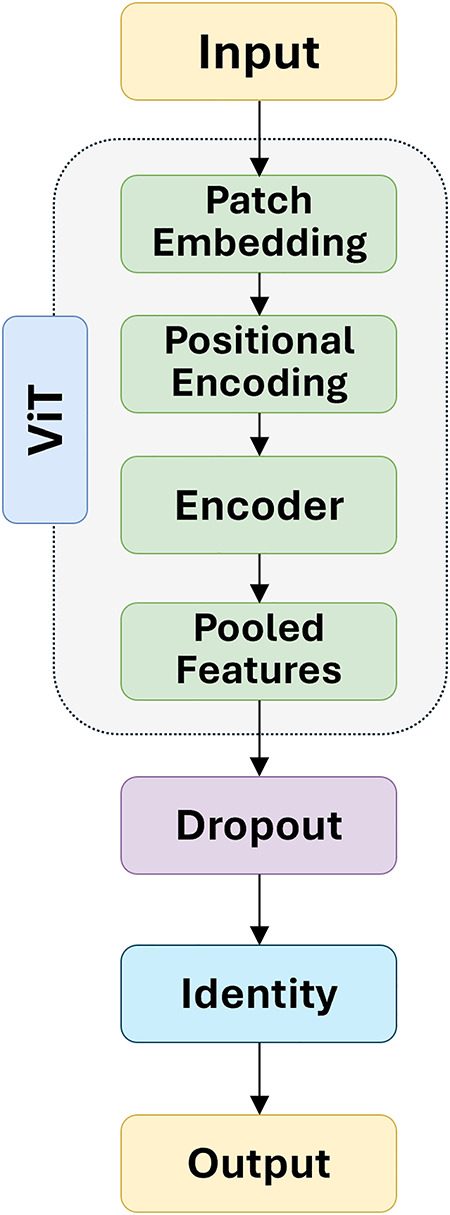
Architecture of ViT.

The overall model, as shown in Fig 3, includes feature extraction which involves using two advanced pre-trained models, DenseNet169 and ViT, along with an AM. These models are chosen for their proven efficiency and effectiveness in image classification tasks [47,48]. DenseNet169, a CNN known for its unique architecture, features dense connectivity where each layer is connected to every other layer in a feed-forward fashion. This design mitigates the vanishing gradient problem, encourages feature reuse, and improves the flow of information and gradients throughout the network. For feature extraction, DenseNet169 retains its feature extraction layers while removing the final classification layer. The model processes input images, resized to 224 × 224 pixels, through a series of convolutional layers that facilitate comprehensive feature learning. The input images are resized to 224 × 224 pixels to ensure consistent dimensions across the dataset, enabling efficient and uniform processing by the model. The output feature maps from the final convolutional block are passed through a global average pooling layer, reducing each feature map to a single value. This produces a fixed-size feature vector of dimension 1664, regardless of the original image dimensions. The ViT introduces a transformer-based architecture as shown in Fig 4, to image classification tasks, which were traditionally dominated by convolutional networks. ViT processes images by dividing them into patches and treating each patch as a “token,” similar to words in natural language processing. Configured to accept images of size 224 × 224 pixels, the ViT model divides each image into 16 × 16 pixel patches, resulting in a sequence of 14 × 14 patches. Each patch is flattened into a vector and embedded into a higher-dimensional space of 1024 dimensions. The model consists of six transformer layers, each with 16 attention heads, allowing it to focus on different parts of the image patches and capture intricate details and spatial relationships. The multi-layer perceptron dimension is set to 2048, providing a robust network for feature learning, with dropout rates of 0.1 applied to prevent overfitting and enhance model performance. After processing through the transformer layers, the model outputs a comprehensive feature vector of dimension 500, representing the entire image.

### Attention Model (AM)

**Fig 5:**
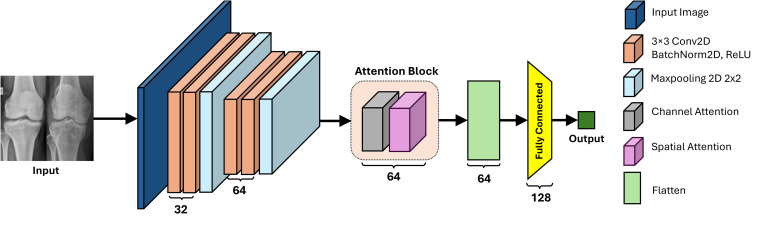
Architecture of the proposed AM.

The AM block is a core component of our model, specifically designed to detect osteoporosis by capturing both spatial and channel-specific information from knee radiographs. It uses multiple convolution layers and attention mechanisms to highlight critical features indicative of osteoporosis, such as cortical thinning, disrupted trabecular patterns, and micro-cracks. By emphasizing key channels that represent these pathological markers and focusing on essential image regions where abnormalities like bone porosity or fractures occur, the AM block is tailored to the unique characteristics of the radiographs. The AM block processes the input image through two parallel attention pathways: one for channel attention and another for spatial attention. The channel attention module selectively recalibrates feature channels by boosting those most relevant for detecting osteoporotic changes, while the spatial attention module aggregates and refines information to pinpoint regions with subtle structural anomalies. The outputs from these two mechanisms are then concatenated with the features from DenseNet169 and ViT, forming a comprehensive feature vector that integrates both global context and fine-grained local details. This integrated representation enhances the model’s discriminative power, leading to more accurate and robust osteoporosis detection. As shown in Fig 5, the block consists of multiple convolutional layers, each followed by ReLU activation functions and max-pooling layers. The first convolutional layer applies 32 filters of size 3 × 3 to the input, producing an output with dimensions (32, 222, 222). The second convolutional layer also uses 32 filters of size 3 × 3, resulting in feature maps with dimensions (32, 220, 220), which are then reduced by a max-pooling layer to dimensions (32, 110, 110). Further layers include a third and fourth convolutional layer, each with 64 filters of size 3 × 3, producing feature maps with dimensions (64, 108, 108) and (64, 106, 106), respectively. Another max-pooling layer reduces these dimensions to (64, 53, 53). The AM incorporates channel and spatial attention mechanisms to improve feature representation. The channel attention mechanism uses adaptive average and max pooling, which compute both the average and maximum values across the spatial dimensions to capture global contextual information and then passes these pooled features through a shared MLP. This shared MLP, a small fully connected network, learns non-linear interactions between channels and generates attention weights that emphasize the most significant channels. The spatial attention mechanism concatenates average-pooled and max-pooled feature maps along the channel axis, followed by a convolutional layer with a kernel size of seven, producing spatial attention weights that highlight important spatial regions. This block effectively captures and enhances relevant features, making it a crucial component of the overall feature extraction process in our model.

Following the feature fusion step, the FC layer is tuned for optimal performance. Extensive experimentation led to the following configuration: The input to the FC layer is a concatenated feature vector of size 2292, combining outputs from DenseNet169 (1664 features), ViT (500 features), and AM (128 features). The first layer reduces the input size from 2292 to 1024 neurons. It includes batch normalization to stabilize learning, a ReLU activation function to introduce non-linearity, and a dropout layer with a rate of 0.5 to prevent overfitting. The second layer further reduces the size from 1024 to 512 neurons, with batch normalization, ReLU activation, and a dropout rate of 0.3. The third layer reduces the size from 512 to 256 neurons, with batch normalization, ReLU activation, and a dropout rate of 0.2. The fourth layer reduces the size from 256 to 128 neurons, with batch normalization, ReLU activation, and a dropout rate of 0.1. The final output layer reduces the size from 128 to 1 neuron, using a sigmoid activation function to produce a binary classification output indicating the presence of osteoporosis or a normal condition. This configuration of layers, channels, and dropout rates was determined to be optimal after extensive experimentation and tuning, ensuring that the model effectively captures and leverages the features extracted from the input images, providing a robust and accurate classification of osteoporosis. The model’s ability to detect osteoporosis is enhanced by combining deep feature extraction with attention mechanisms. The convolutional layers capture features from low to high levels across the radiograph, while the attention mechanisms selectively enhance ROIs. The FC network then integrates and processes these features to produce an accurate classification of osteoporosis presence or absence. By tailoring the model specifically to this dataset, it becomes highly sensitive to the subtle variations in BMD and structures critical for identifying osteoporosis.

### Comparisons with SOTA and ablation study

To assess the performance of our proposed approach, we independently evaluated the performances of several deep-learning models to identify the most effective approach for osteoporosis detection using KOP dataset. Initially, each model is tested individually to determine its strengths and weaknesses. The assessed SOTA models included the VGG19 [49], EfficientNetB0 [46], ResNet50 [24], InceptionV3 [50], DenseNet169 [13], and ViT models [14] and we also compared our model with recent top models like ConvNext-V2 [51], RDNet [52], and MetaFormer [53], we retrained these three models on our dataset rather than using pre-trained weights to ensure a fair comparison with the existing top models.. All models were trained and evaluated using identical dataset splits and performance metrics to ensure a fair comparison. Additionally, we compared our model with KONet, a recent approach for osteoporosis detection on the KOP dataset. To ensure consistency, we re-implemented KONet following its original training protocol, using an 80% training, 10% validation, and 10% testing split.

We also performed an ablation study by removing some components to see the effectiveness of each component. In this ablation study, individual components of the model were systematically removed to assess their contributions to overall performance. We designed multiple variations by combining different components of the model, including Variation A, Variation B, Variation C, Variation D, and finally, the proposed BONE-Net. By selectively omitting each component one by one, we were able to quantify the impact of each component on the model’s overall performance. This analysis provided valuable insights into which parts of the network were essential for robust feature extraction and accurate classification.

For the experiments, several performance metrics were used to evaluate the models. These metrics thoroughly assess the effectiveness of the models in detecting osteoporosis. Table 1 summarizes the performance metrics employed, while Table 2 provides detailed information on the parameters used in our analysis. We have also visualized our model’s decision-making process using Grad-CAM, which provides a window into the areas that drive its predictions.

**Table 1:** Summary of the performance metrics used.

Metrics	Quantity
**Sensitivity**	TPR = TP/ (TP + FN)
**Specificity**	SPC = TN/ (FP + TN)
**Precision**	PPV = TP/ (TP + FP)
**FPR**	FPR = FP/ (FP + TN)
**Accuracy**	ACC = (TP + TN)/ (P + N)
**F1 Score**	F1 = 2TP/ (2TP + FP + FN)

**True Positives (TP)**: Correctly identified osteoporotic cases.

**False Positives (FP):** Non-osteoporotic cases incorrectly identified as osteoporotic.

**True Negatives (TN):** Correctly identified non-osteoporotic cases.

**False Negatives (FN)**: Osteoporotic cases incorrectly identified as non-osteoporotic.

**Loss** represents the prediction error of the model, with lower values indicating better performance.

**Table 2:** Summary of the parameters used.

Component	Description
**Development Environment**	Python, Pytorch, Jupyter Notebook
**Processor**	Intel® Xeon(R) Silver 4210R CPU @ 2.40GHz × 40
**RAM**	256 GB
**Operating System**	Linux
**GPU**	NVIDIA GeForce RTX 3090
**Input image dimensions**	224 × 224 × 3 pixels
**Epochs**	50

## Results

This section presents the performance evaluation of our proposed model alongside various baseline and SOTA deep-learning architectures on the KOP dataset. The results highlight each model’s strengths across key clinical metrics, including sensitivity, specificity, accuracy, F1-score, precision, and false positive rate (FPR). This comparative analysis aims to identify the most effective approach for osteoporosis detection in a medical imaging context.

ViT demonstrated exceptional sensitivity at 0.9444, indicating its reliability and accuracy, which is a crucial factor in medical diagnostics where false positives can lead to unnecessary treatments. InceptionV3 led to an overall accuracy of 0.8158, making it highly effective for correctly classifying both positive and negative instances. It also exhibited the lowest loss of 0.5885, suggesting that it had the fewest errors per sample, which is beneficial for reliable predictions in clinical applications. Among these models, DenseNet169 notably achieved the highest accuracy of 0.8289 and an F1 score of 0.8354, effectively balancing precision. This balance is essential in medical diagnostics, as identifying true positives is critical for applications where failing to detect a positive case could have serious consequences. The AM had the highest specificity of 0.8333, effectively minimizing false positives, which is crucial for reducing unnecessary alarms in clinical settings. VGG19 offers superior precision (0.9211), emphasizing its accuracy in identifying positive instances, thereby reducing the chances of false positives. Additionally, it had the lowest FPR (0.1667), further underscoring its efficiency in minimizing incorrect positives, where both false positives and false negatives have significant implications.

Furthermore, EfficientNetB0 achieved an accuracy of 0.8026, sensitivity of 0.7347, specificity of 0.8774, FPR of 0.2643, and precision of 0.9259, demonstrating a strong balance between identifying positive cases and minimizing false positives. ResNet50 showed an accuracy of 0.7632, sensitivity of 0.7083, specificity of 0.8947, FPR rate of 0.2917, and precision of 0.8571, indicating solid performance with slightly lower accuracy but good specificity. InceptionV3, as noted, reached 0.8158 accuracy with sensitivity and specificity of 0.7609 and 0.9211 respectively, reinforcing its strength in balanced classification.

BONE-Net demonstrated high specificity (0.9474) and precision (0.9286), indicating its effectiveness in reducing false positives in osteoporosis detection. RDNet and MetaFormer showed comparatively higher sensitivity values (0.8158 and 0.8182), which could be advantageous for detecting positive cases. These differences highlight the varying strengths of each model across key performance metrics. ConvNeXt-V2, with high specificity (0.7895) and competitive precision (0.7714), presents a balanced option but still falls short of BONE-Net in terms of overall accuracy (0.7500 vs. 0.8611). Additionally, our model also achieves the lowest loss of 0.5742 which indicates better model stability and convergence and are critical for generalization in real-world applications. This comparison highlights BONE-Net’s potential as a promising approach for medical image classification and clinical deployment, especially in environments, where minimizing false positives and achieving high accuracy are key priorities.

DenseNet169 demonstrated superior performance in comparison to others making it the best choice for integration into the final model architecture, we selected DenseNet169 to enhance the effectiveness and reliability of the proposed model. This strategic choice was aimed at using DenseNet169’s strengths to improve our models’ overall capability to detect osteoporosis with high accuracy and balanced performance metrics, thereby ensuring more accurate and reliable diagnostics in clinical settings. DenseNet169 resulted in a higher performance than the other models. Specifically. The overall results of the proposed model compared to different baseline models are summarized in Table 3.

**Table 3:** Summary of the overall results compared to baseline models.

Models	Sensitivity	Specificity	Precision	FPR	Accuracy	F1 Score	Loss
**CovNext-V2** [40]	0.7105	0.7895	0.7714	0.2105	0.7500	0.7397	0.6385
**RDNET** [52]	0.8158	0.7105	0.7381	0.2895	0.7632	0.7419	0.6382
**MetaFormer** [53]	0.8182	0.7442	0.7105	0.2558	0.7763	0.7606	0.6251
**VGG19** [49]	0.8889	0.7143	0.9211	0.2857	0.7763	0.8046	0.6294
**EfficientNetB0** [46]	0.9259	0.7347	0.8774	0.2643	0.8026	0.8276	0.6029
**ResNet50** [24]	0.8571	0.7083	0.8947	0.2917	0.7632	0.7907	0.6236
**InceptionV3** [50]	0.9000	0.7609	0.9211	0.2391	0.8158	0.8333	0.5885
**Densenet169** [13]	0.8571	0.8049	0.8684	0.1951	0.8289	0.8354	0.5892
**ViT** [14]	**0.9444**	0.6852	0.7782	0.3148	0.7500	0.8043	0.6498
**Ours (BONE-Net)**	0.8293	**0.9474**	**0.9286**	**0.0526**	**0.8611**	**0.8387**	**0.5742**

**Fig 6:**
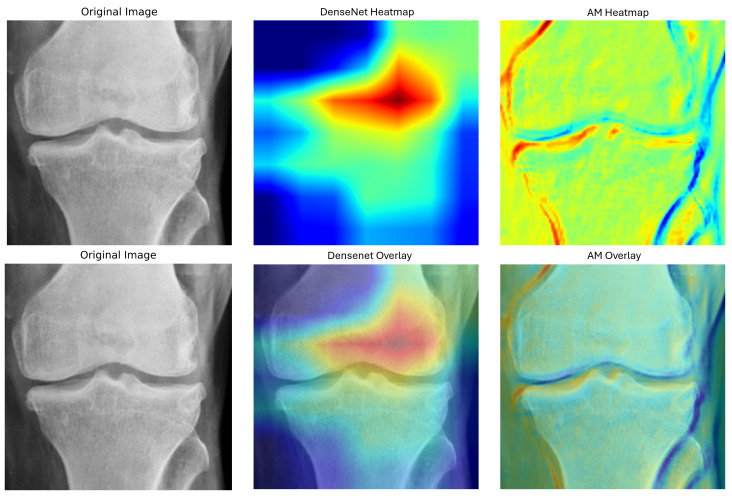
Grad-CAM visualization of the CNN-based components of BONE-Net.

**Fig 7:**
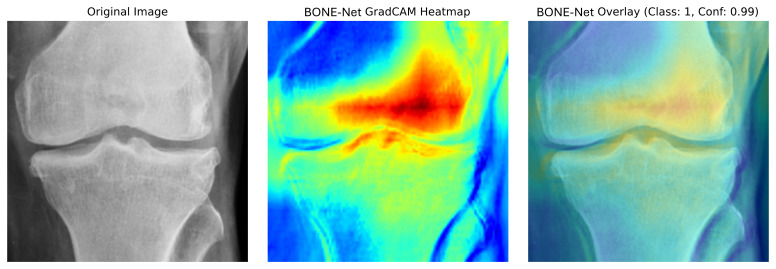
Grad-CAM visualization of BONE-Net.

Simultaneously, the ViT was incorporated for its robust capability to capture global features through its Transformer-based architecture. Although the standalone ViT model achieved an exceptional sensitivity of 0.9444 demonstrating its strength in accurately identifying non-osteoporotic cases it exhibited lower specificity of 0.6852 and overall accuracy of 0.7500 compared to DenseNet169. This indicates that while ViT is highly effective in detecting true positives, it tends to produce more false positives. To address this, our designed AM was developed to enhance feature extraction by capturing additional spatial and channel-specific information. Our BONE-Net, which passed through a rigorously tuned fully connected (FC) layer to integrate and process the comprehensive feature set for final classification, achieved the highest accuracy of 0.8611, specificity of 0.9474, FPR of 0.0526, and an F1 score of 0.8387. These results demonstrate the model’s superior performance in accurately distinguishing between osteoporotic and normal cases, highlighting its effectiveness in clinical applications. Furthermore, the Grad-CAM visualization, as shown in Figs 6 and 7, provides a window into the areas that drive the model’s predictions, offering valuable insights into its decision-making process.

In Fig 6, the DenseNet component clearly shows that the model focuses on key bony structures, emphasizing regions that are critical for assessing osteoporosis. Meanwhile, the visualization of the AM highlights additional, more refined regions of interest, further corroborating the anatomical relevance of the model’s focus. Together, these visualizations enhance the explainability of our approach by confirming that both DenseNet and the AM contribute significantly to identifying the most informative features in the images. This, in turn, bolsters the clinical credibility and interpretability of our osteoporosis detection system. Fig 7 visualizes the combined Grad-CAM for the overall BONE-Net model, providing an integrated view that reflects the synergistic effect of the fused components. The heatmaps overlaid on the radiographs clearly illustrate the specific anatomical regions that the model prioritizes during the decision-making process. This focused attention on clinically relevant structures not only confirms the model’s capability to target essential diagnostic areas but also supports its interpretability and reliability in practical clinical settings. Collectively, these visual explanations serve to validate our model’s focus on medically meaningful features, enhancing trust and acceptance of the automated detection system among clinicians.

### Confusion matrix

**Fig 8:**
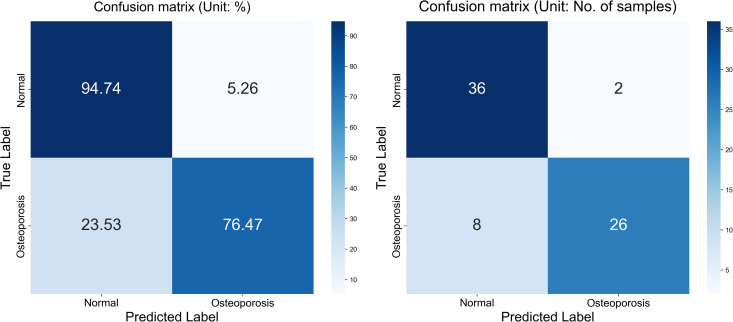
Confusion matrix of BONE-Net.

**Fig 9:**
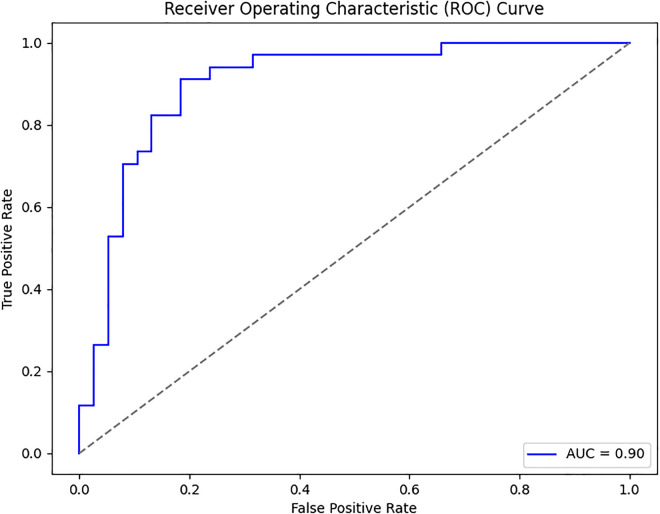
The AUC-ROC curve visualizes the performance of the model by plotting the TPR against the FPR at various threshold settings. The curve represents the trade-off between sensitivity (recall) and specificity, with the diagonal dashed line acting as a reference that represents random guessing. The model achieves an AUC of 0.92, indicating strong discriminative ability and suggesting that the model is effective at distinguishing between positive and negative classes. The closer the curve follows the left and top borders of the plot, the better the model’s performance.

**Fig 10:**
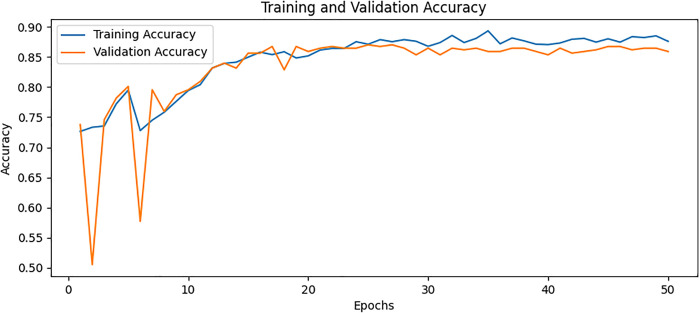
Training and validation accuracy for BONE-Net.

**Fig 11:**
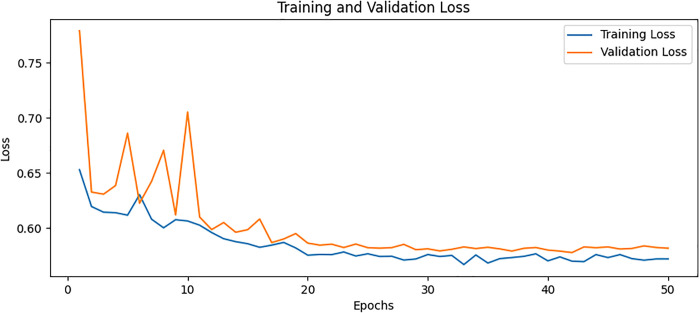
Training and validation loss for BONE-Net.

The confusion matrix presented in Fig 8 for the proposed model displays the relationship between actual values and predicted values for the classification of two classes. The plot on the left shows that TP is 36, FP is 2, FN is 8, and TN is 26. The plot on the right shows these values as percentages corresponding to a TP of 94.74% with FP of 5.26% only TN of 76.47% and FN of 23.53%. The values are divided into two portions: normal and osteoporotic. Following the confusion matrix, we analyze the Area Under the Receiver Operating Characteristic AUC-ROC curve as illustrated in Fig 9, which graphically represents the model’s true positive rate against the FPR. This curve provides insights into the model’s performance across different decision thresholds, highlighting the model’s ability to distinguish between the two classes across a range of sensitivities and specificities. Additionally, our model achieved the lowest loss value of 0.5742, outperforming all other evaluated SOTA methods. Compared to KONet, our model demonstrated superior performance. The comparative results are summarized in Table 4. Figs 10 and 11 show the training and validation accuracies and losses of our model, respectively, illustrating its convergence and stability throughout the training process. These significant improvements highlight the effectiveness of our approach, demonstrating its potential for accurate osteoporosis detection and potentially enhancing clinical outcomes.

**Table 4:** Comparison with KONet.

Metrics	BONE-Net	KONet [36]
**Sensitivity**	**0.8293**	0.8110
**Specificity**	**0.9474**	0.7556
**Precision**	**0.9286**	0.7105
**FPR**	**0.0526**	0.2444
**Accuracy**	**0.8611**	0.8026
**F1 Score**	**0.8387**	0.7826
**Loss**	**0.5742**	0.5983

### Ablation study

To further understand the contributions of each model component, we conducted an ablation analysis to systematically test the impact of DenseNet169, ViT, and AM individually and in various combinations. – show the training and validation accuracy for Variation A, Variation B, Variation C, and Variation D, respectively. These plots illustrate how different architectural components impact model performance. By systematically removing specific elements, such as AM, feature fusion, and additional processing layers, we observe variations in learning stability, convergence rates, and final accuracy. The full model, which integrates DenseNet, ViT, and AM, demonstrates the highest accuracy and the most stable training curve, indicating that each component contributes to improved feature extraction and classification. In contrast, models with reduced architecture exhibit lower accuracy and more fluctuations in validation performance, highlighting the significance of each component in enhancing osteoporosis detection. The overall combinations and results of the ablation study are provided in Table 5 and 6, respectively. The ablation study provided the following insights.

**Table 5:** Components of variations in the ablation study.

VARIATIONS	DenseNet169	ViT	AM	# of Params
**VARIATION A**	✔	✔	**×**	67M
**VARIATION B**	**×**	**×**	✔	23M
**VARIATION C**	✔	**×**	✔	89M
**VARIATION D**	**×**	✔	✔	87M
**Ours (BONE-Net)**	✔	✔	✔	90M

**Table 6:** Results of the ablation study.

VARIATIONS	Sensitivity	Specificity	Precision	FPR	Accuracy	F1 Score	Loss
**VARIATION A**	0.8235	0.7619	0.8421	0.2381	0.7895	0.8000	0.5993
**VARIATION B**	0.7778	0.8333	0.7368	0.1667	0.8056	0.8108	0.6020
**VARIATION C**	0.8438	0.7500	0.8684	0.2500	0.7895	0.8049	0.6026
**VARIATION D**	0.7297	0.8000	0.7368	0.2000	0.7639	0.7671	0.6121
**Ours (BONE-Net)**	**0.8293**	**0.9474**	**0.9286**	**0.0526**	**0.8611**	**0.8387**	**0.5742**

**Fig 12:**
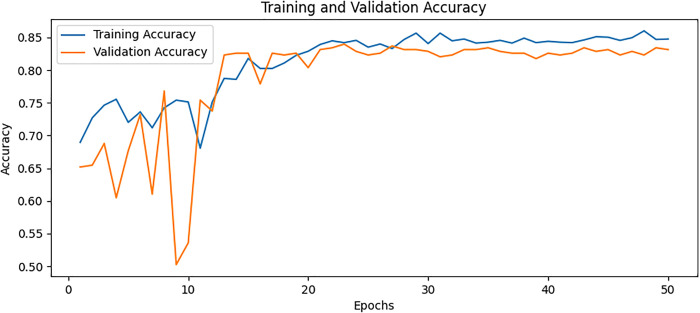
Training and validation accuracy for Variation A.

**Fig 13:**
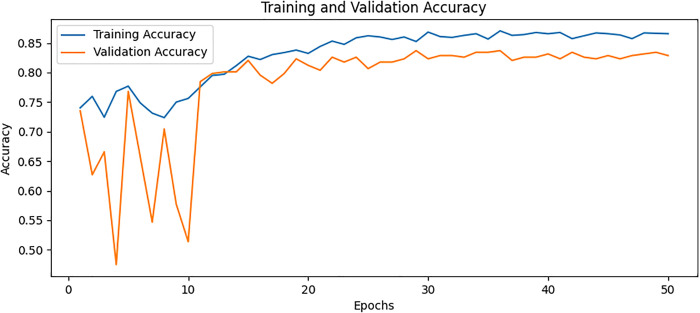
Training and validation accuracy for Variation B.

**Fig 14:**
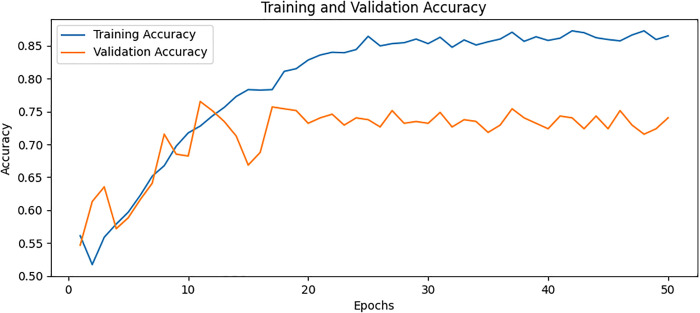
Training and validation accuracy for Variation C.

**Fig 15:**
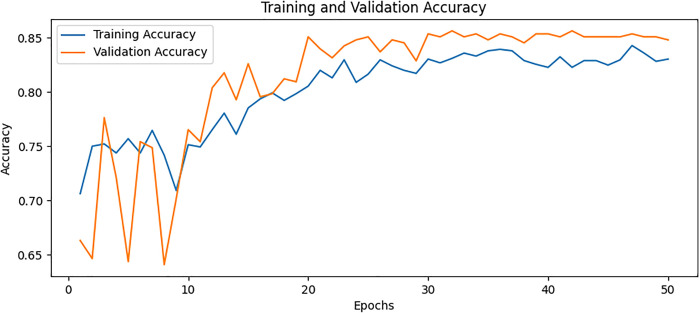
Training and validation accuracy for Variation D.

### Individual model performance and combinations

DenseNet169 and ViT, when evaluated individually, demonstrated strong capabilities for feature extraction, with DenseNet169 achieving an accuracy of 0.8289 and ViT achieving the highest sensitivity of 0.9444. The AM test showed better performance with the highest specificity of 0.8333 and an accuracy of 0.8056. Notably, the AM achieved an FPR of 0.1667 and a loss of 0.6020. The accuracy of ViT in the individual test was 0.7500.

We further investigated the AM’s contribution by integrating it with both DenseNet169 and ViT. Integrating DenseNet169 with AM improved sensitivity and precision, achieving an accuracy of 0.7895. Similarly, combining ViT with AM balanced specificity and reduced the FPR, though it did not surpass that of the Variation C combination. When AM was integrated with ViT, accuracy increased to 0.7639, indicating the effectiveness of AM. The Variation B combination reached an accuracy of 0.7895, highlighting the complementary strengths of these models.

Notably, the integration of the AM with ViT improved overall performance: although this integration resulted in a modest reduction in sensitivity compared to standalone ViT, it substantially improved specificity from 0.6852 to 0.8000 and lowered the FPR from 0.3148 to 0.2000. This balanced performance is critical in clinical diagnostics, where minimizing false positives is essential to avoid unnecessary follow-up tests and treatments.

Integrating DenseNet169, ViT, and AM in the final model resulted in a significant performance boost, achieving the highest accuracy (0.8611) and the lowest loss (0.5742). This model also outperformed all baseline models across all key metrics, highlighting the effectiveness of combining DenseNet’s local feature extraction, ViT’s global representation, and AM’s spatial focus. The ablation analysis confirms that our integrated architecture, DenseNet169’s local feature extraction, ViT’s global contextual understanding, and the AM’s focused enhancement of critical features, work synergistically to provide a more robust and clinically applicable detection system.

## Discussion

In the evaluation of various models for detecting osteoporosis on the KOP dataset, each model showed distinct strengths across critical performance metrics. DenseNet169 and ViT outperformed other models individually. By integrating these models with the designed AM, we propose a combined model called BONE-Net that achieves the highest performance in terms of accuracy, F1 score, and loss. The extensively tuned FC layer plays a crucial role in integrating the features from the models, contributing to the superior performance of the model. The results show that our approach effectively combines the strengths of DenseNet169, ViT, and the AM. DenseNet169’s dense connectivity captures local features efficiently, while ViT excels at capturing global features. The AM block enhances feature extraction by incorporating both spatial and channel-specific information. The FC layer integrates these features into a comprehensive representation, improving classification accuracy.

The fusion of DenseNet169, ViT, and AM underlines their strengths to enhance osteoporosis detection from knee X-ray images. This fusion approach not only combines local and global information for a robust diagnostic process but also addresses the individual weaknesses of each model. For example, while DenseNet169 might capture detailed local features, ViT extends this analysis by integrating broader patterns that might otherwise be missed and, hence, captures global features. The AM further ensures that the network prioritizes these crucial areas in the final analysis, thus enhancing both the sensitivity and specificity of the diagnosis. This integration allows architecture to benefit from both local and global information, address individual model limitations, and enhance diagnostic accuracy. Moreover, this fusion strategy improves the performance of the model on test data, reduces overfitting risks and ensures robust performance across diverse clinical scenarios. The integration of DenseNet169, ViT, and AM within our framework effectively utilizes the distinct advantages of each method to develop an integrated system that is not only computationally efficient but also exceptionally proficient in the early detection of osteoporosis. This approach optimizes performance across vital diagnostic metrics, promising significant improvements in the accuracy and reliability of osteoporosis screening in clinical settings. This innovative approach is poised to advance the standard of care for osteoporosis management.

The high specificity of BONE-Net is clinically important because it reduces FPR, thereby avoiding over-diagnosis and unnecessary interventions. The comparison between BONE-Net and ConvNext-V2, RDNet, and MetaFormer highlights the trade-offs between different architectures and their implications for clinical applications. ConvNeXt-V2 offers a balanced option in specificity and precision but is less accurate overall compared to BONE-Net. BONE-Net’s lower loss indicates improved model stability and better overall performance, which are critical for practical deployment. The choice of DenseNet169 was driven by its balanced performance across accuracy, precision, and sensitivity. ViT excels at capturing global features but tends to generate more false positives, highlighting its strengths and limitations. The AM enhances feature extraction by focusing on spatial and channel-specific information, thereby improving clinical applicability by reducing false positives. The fusion strategy combining DenseNet169, ViT, and the AM addresses individual model weaknesses and improves robustness across diverse clinical scenarios. The Grad-CAM heatmap visualizations demonstrate the model’s ability to focus on anatomically relevant regions, supporting explainability and clinical credibility. The DenseNet component appears to concentrate more specifically on the central femoral condyle, while the AM block shows greater sensitivity to joint spaces and bone margins. When overlaid on the original images, these visualizations confirm that our model appropriately targets anatomically relevant structures critical for osteoporosis assessment, rather than irrelevant background features. The complementary attention patterns between the components validate our ensemble approach, as each network element captures slightly different but clinically significant bone characteristics. These visualizations confirm that BONE-Net effectively prioritizes the most informative areas, enhancing both interpretability and clinical utility.

Additionally, our approach aligns with recent advancements in ensemble learning, such as KONet, which integrates various architectures for knee osteoporosis classification. KONet enhances performance by effectively combining features from multiple models, leading to improvements in classification accuracy, sensitivity, and specificity. Compared to KONet, BONE-Net demonstrated superior performance across all key metrics. While KONet leverages ensemble strategies to optimize its accuracy and sensitivity, our model outperformed KONet with higher sensitivity, specificity, precision, and accuracy. Moreover, our model exhibited a lower false-positive rate and a more favorable loss value. This marked improvement suggests that our integrated approach, which effectively combines DenseNet169, ViT, and the AM, provides a more robust and accurate solution for detecting osteoporosis. The overall performance of BONE-Net suggests strong potential for clinical deployment and early detection of osteoporosis, contributing to more reliable and timely diagnosis.

The ablation study provided critical insights into the contribution of each model component to overall performance. By systematically removing DenseNet169, ViT, and the AM, we were able to quantify the impact of each element on sensitivity, specificity, accuracy, and loss. The AM significantly contributes to enhancing model performance, particularly in terms of specificity and false positive reduction. When evaluated individually (Variation B), the AM achieved the highest accuracy (0.8108), highest specificity (0.8333) and the lowest FPR (0.1667) among the partial configurations, indicating its ability to effectively reduce FPs, a crucial requirement in clinical diagnostics. Additionally, its integration with DenseNet169 (Variation C) and ViT (Variation D) further improved the performance, demonstrating the AM’s capacity to refine feature representation. In the full BONE-Net configuration, the inclusion of AM led to the best overall results improving the specificity, Accuracy, FPR, Loss and other important performance metrics confirming that this module plays a vital role in focusing on discriminative spatial and channel features, thereby enhancing diagnostic reliability and clinical applicability. The study revealed that the full integration of all components consistently achieved the best results, while the removal of any individual component led to a noticeable degradation in performance. These findings underscore the complementary roles of dense connectivity, global feature extraction, and the enhanced feature refinement provided by the AM, confirming that the synergistic interaction among DenseNet169, ViT, and the AM is essential for robust and accurate osteoporosis detection.

The enhanced performance of BONE-Net suggests significant potential for accurate osteoporosis detection, crucial for timely intervention to prevent fractures and improve patient outcomes. The model’s high accuracy, sensitivity, and specificity ensures reliable identification of osteoporosis, making it a valuable tool for clinical applications. This could assist healthcare professionals in making more informed decisions, ultimately reducing the incidence and impact of osteoporotic fractures.

Despite the promising results, our work has several limitations. The dataset used for training and evaluation consists of a relatively small number of images (372), which may limit the model’s generalizability to larger and more diverse datasets. Additionally, the dataset focuses primarily on the KOP dataset, restricting the model’s applicability to other anatomical sites commonly affected by osteoporosis, such as the hip or spine. Another limitation is that the model was trained using only image data, excluding important clinical factors such as patient age, gender, and BMD, which could enhance predictive accuracy and provide a more comprehensive assessment of osteoporosis risk. Future research should address these limitations by expanding the dataset to include images from other anatomical sites affected by osteoporosis, which would improve the model’s robustness and clinical applicability. Additionally, incorporating multi-modal data such as clinical history, patient demographics, and BMD measurements into the model could provide a more comprehensive assessment of a patient’s osteoporosis risk.

## Conclusion

In this study, we developed a hybrid deep-learning approach for osteoporosis detection using KOP dataset. Our methodology combines the feature extraction capabilities of two SOTA pre-trained models, DenseNet169 and ViT, with a custom-designed AM that incorporates channel and spatial attention mechanisms. This integrated approach achieved superior performance in classifying osteoporotic conditions. By combining DenseNet169’s robust feature extraction with ViT’s global feature capture and augmenting this with the spatial and channel-wise AM block, we provided a comprehensive image representation that significantly improved classification performance. Our results demonstrate the effectiveness of integrating these advanced models with custom-designed blocks for medical image analysis, offering a notable advancement over existing SOTA techniques. This method shows promise for clinical applications in osteoporosis diagnosis, facilitating earlier detection and more targeted interventions, which could help reduce the morbidity and mortality associated with osteoporotic fractures. Future research may focus on further refining the model, utilizing larger datasets, and exploring its application to other bone-related conditions for broader clinical adoption.
